# Pancreatic Alpha-Cells Contribute Together With Beta-Cells to CXCL10 Expression in Type 1 Diabetes

**DOI:** 10.3389/fendo.2020.00630

**Published:** 2020-09-15

**Authors:** Laura Nigi, Noemi Brusco, Giuseppina E. Grieco, Giada Licata, Lars Krogvold, Lorella Marselli, Conny Gysemans, Lut Overbergh, Piero Marchetti, Chantal Mathieu, Knut Dahl Jørgensen, Guido Sebastiani, Francesco Dotta

**Affiliations:** ^1^Diabetes Unit, Department of Medicine, Surgery and Neurosciences, University of Siena, Siena, Italy; ^2^Fondazione Umberto Di Mario, c/o Toscana Life Sciences, Siena, Italy; ^3^Faculty of Odontology, University of Oslo, Oslo, Norway; ^4^Faculty of Medicine, University of Oslo, Oslo, Norway; ^5^Department of Clinical and Experimental Medicine, University of Pisa, Pisa, Italy; ^6^Clinical and Experimental Endocrinology (CEE), Katholieke Universiteit Leuven (KU LEUVEN), Leuven, Belgium; ^7^Division of Paediatric and Adolescent Medicine, Oslo University Hospital, Oslo, Norway; ^8^Tuscany Centre for Precision Medicine (CReMeP), Siena, Italy

**Keywords:** type 1 diabetes, pancreas, alpha-cells, chemokines, CXCL10

## Abstract

C-X-C Motif Chemokine Ligand 10 (CXCL10) is a pro-inflammatory chemokine specifically recognized by the ligand receptor CXCR3 which is mostly expressed in T-lymphocytes. Although CXCL10 expression and secretion have been widely associated to pancreatic islets both in non-obese diabetic (NOD) mice and in human type 1 diabetic (T1D) donors, the specific expression pattern among pancreatic endocrine cell subtypes has not been clarified yet. Therefore, the purpose of this study was to shed light on the pancreatic islet expression of CXCL10 in NOD, in C57Bl/6J and in NOD-SCID mice as well as in human T1D pancreata from new-onset T1D patients (DiViD study) compared to non-diabetic multiorgan donors from the INNODIA European Network for Pancreatic Organ Donors with Diabetes (EUnPOD). CXCL10 was expressed in pancreatic islets of normoglycaemic and new-onset diabetic NOD mice but not in C57Bl/6J and NOD-SCID mice. CXCL10 expression was increased in pancreatic islets of new-onset diabetic NOD mice compared to normoglycaemic NOD mice. In NOD mice, CXCL10 colocalized both with insulin and glucagon. Interestingly, CXCL10-glucagon colocalization rate was significantly increased in diabetic vs. normoglycaemic NOD mouse islets, indicating an increased expression of CXCL10 also in alpha-cells. CXCL10 was expressed in pancreatic islets of T1D patients but not in non-diabetic donors. The analysis of the expression pattern of CXCL10 in human T1D pancreata from DiViD study, revealed an increased colocalization rate with glucagon compared to insulin. Of note, CXCL10 was also expressed in alpha-cells residing in insulin-deficient islets (IDI), suggesting that CXCL10 expression in alpha cells is not driven by residual beta-cells and therefore may represent an independent phenomenon. In conclusion, we show that in T1D CXCL10 is expressed by alpha-cells both in NOD mice and in T1D patients, thus pointing to an additional novel role for alpha-cells in T1D pathogenesis and progression.

## Introduction

Type 1 diabetes (T1D) is an autoimmune disease, characterized by a progressive destruction of pancreatic insulin-producing beta-cells driven by autoreactive T-lymphocytes (1) and leading to chronic hyperglycemia and to the development of chronic complications (2).

C-X-C Motif Chemokine Ligand 10 (CXCL10) is a pro-inflammatory chemokine secreted by a wide spectrum of cells. It is involved in multiple mechanisms and reported to have pleiotropic effects, including immune cell migration and attraction to inflammation sites, angiogenesis, and cancer cell growth (3, 4). CXCL10 is produced by pancreatic islet cells upon inflammatory stress (5) and is specifically recognized by C-X-C Motif Chemokine Receptor 3 (CXCR3) which is expressed by activated T-lymphocytes and other immune cells (4, 6). Several reports demonstrated that CXCL10 plays an important role in the natural history of T1D mainly through the attraction of autoreactive T-lymphocytes to the islets, thus leading to the subsequent destruction of pancreatic beta-cells (6–12). Of note, although still debated, CXCL10 has been proposed as a possible therapeutic target, supported by several studies showing the beneficial effects of CXCL10 inhibition (13).

In animal models, transgenic overexpression of CXCL10 in beta-cells, coupled to the induction of T1D through lymphocytic choriomeningitis virus (LCMV) infection, accelerated the autoimmune process by enhancing the migration of antigen-specific lymphocytes (6, 7, 10). Of note, neutralization of CXCL10 reduced the occurrence of the disease by affecting lymphocyte migration to the pancreatic islets and by enhancing beta-cell proliferation (14–18). Similar results, although more controversial, were reported in CXCR3-deficient mouse models or upon antagonistic blockade of CXCR3, leading to delayed insulitis and diabetes onset (19). Therefore, CXCL10:CXCR3-based pancreatic immune cell trafficking has been reported as an important component in the natural history of T1D.

In man, increased CXCL10 levels have been detected in serum of T1D patients compared to non-diabetic subjects and to type 2 diabetic (T2D) individuals, thus indicating a relationship between this phenomenon and autoimmune diabetes (20–23). Of note, CXCL10 levels were positively correlated with the numbers of autoreactive CD4 T cells and negatively associated with T1D duration and age at disease onset, suggesting important implications for CXCL10 in autoimmune diabetes progression and severity (21).

Using immunohistochemistry and immunofluorescence, we and others have previously demonstrated that CXCL10 expression is increased in pancreatic islets of T1D vs. non-diabetic donors (24–26). However, the exact CXCL10 expression pattern in pancreatic islet endocrine cell subsets has not been addressed.

Here, by using immunohistochemical fluorescence and confocal imaging, we aimed at elucidating the CXCL10 expression pattern among pancreatic islet endocrine cells in T1D through the analysis of pancreas samples from NOD, C57Bl/6J and NOD-SCID mice as well as from new-onset T1D patients (DiViD study) and non-diabetic multiorgan donors from the INNODIA European Network for Pancreatic Organ Donors with Diabetes (EUnPOD).

## Materials and Methods

### Animals

C57Bl/6J, NOD-SCID, and NOD mice were housed and inbred in the animal facility of Katholieke Universiteit Leuven (KU Leuven, Leuven, Belgium) as previously described (27). All animal procedures were performed in accordance with the NIH guidelines for the care and use of laboratory animals and protocols were approved by the Ethics Committees of the KU Leuven. NOD female mice used in this study were screened for the onset of diabetes by evaluating urine glucose levels (Diastix Reagent Strips; Bayer, Leverkusen, Germany) and venous blood glucose levels (Accu-Chek; Roche Diagnostics, Vilvoorde, Belgium). Mice were diagnosed as diabetic when they had glycosuria and two consecutive blood glucose measurements exceeding 200 mg/mL. Pancreatic sections used for histological analysis were collected from 8-week-old C57Bl/6J (*n* = 4), 15- to 20-week-old C57Bl/6J (*n* = 3), 2- to 3-week-old NOD (Non-Obese Diabetic)-SCID (severe combined immunodeficient) (*n* = 4), 20-week-old NOD-SCID (*n* = 3), 20- to 22-week-old normoglycaemic NOD (*n* = 4) and 12- to 21-week-old new-onset diabetic NOD mice (*n* = 4).

### Human Donors

Human pancreatic sections analyzed in this study were collected from two different cohorts of subjects.

#### INNODIA EUnPOD Cohort

Following acquisition of informed research consent, pancreata (*n* = 3) were obtained from brain-dead multiorgan donors within the European Network for Pancreatic Organ Donors with Diabetes (EUnPOD) (2), a project launched in the context of the INNODIA consortium (www.innodia.eu). Whole pancreata were processed following standardized procedures at University of Pisa. Formalin fixed paraffin embedded (FFPE) pancreatic tissue sections were obtained from *n* = 3 non-diabetic and islet-autoantibodies negative donors.

#### DiViD Cohort

Following the acquisition of appropriate consents, 6 new-onset T1D patients underwent pancreatic biopsy by adopting laparoscopic pancreatic tail resection, in the context of the Diabetes Virus Detection (DiViD) study (28). The pancreatic tissue was processed for multiple purposes including FFPE processing (28). From each patient included in the DiViD study, we analyzed two pancreatic sections from separate parts of the pancreas tail for CXCL10-INS-GCG staining and one pancreatic section for CXCL10-INS-CD45 staining. Collection of pancreatic tissue in the DiViD study was approved by the Norwegian Governments Regional Ethics Committee. Written informed consent was obtained from all individuals with type 1 diabetes after they had received oral and written information from the diabetologist and the surgeon separately (28). INNODIA EUnPOD multiorgan donors' pancreata were obtained with approval of the local Ethics Committee at the University of Pisa.

Clinical characteristics of EUnPOD donors and of DiViD T1D subjects are reported in Table 1.

**Table 1:** Demographics and main clinical parameters of T1D and non-diabetic donors.

	**Case ID**	**Gender**	**Age**	**Disease duration (weeks)**	**Cause of death**	**IA**	**GADA**	**IA−2A**	**ZnT8A**
Non-diabetic (EUnPOD)	20171031	F	54	n/a	Cardiovascular disease	n/a	neg	neg	neg
	20171114	M	49	n/a	Cardiovascular disease	n/a	neg	neg	neg
	20171118	M	39	n/a	Trauma	n/a	neg	neg	neg
Type 1 diabetic (DiViD)	DiViD-1	F	25	4	n/a	pos	pos	pos	pos
	DiViD-2	M	24	3	n/a	pos	neg	pos	pos
	DiViD-3	F	34	9	n/a	pos	neg	pos	pos
	DiViD-4	M	31	5	n/a	pos	pos	neg	pos
	DiViD-5	F	24	5	n/a	pos	pos	neg	pos
	DiViD-6	M	35	5	n/a	pos	neg	neg	neg

### Immunofluorescence Analysis of Mouse and Human Pancreatic Sections

Formalin-Fixed Paraffin Embedded (FFPE) pancreatic sections obtained from mouse and human pancreata were analyzed using single or triple immunofluorescence and confocal imaging analysis in order to simultaneously evaluate the expression of CXCL10, insulin, glucagon or CXCL10, insulin, and CD45.

#### Mouse Pancreatic Sections

After deparaffinization and rehydration through alcohol series, pancreatic sections (5 μm thickness) were incubated with Tris-buffered saline (TBS) supplemented with 3% bovine serum albumin (BSA, Sigma-Aldrich, St. Louis, MO, USA) to reduce non-specific reactions. Antigen retrieval was performed using 10 mM citrate buffer pH 6.0 in microwave (600 W) for 10 min. Sections were incubated with polyclonal rabbit anti-murine CXCL10 [dilution 1:25, cat. 500-P129, Peprotech, Rocky Hill, NJ, USA; purified by affinity chromatography employing immobilized mCXCL10 matrix from sera of rabbits pre-immunized with highly pure (>98%) recombinant mCXCL10] (29), polyclonal guinea pig anti-insulin (dilution 1:500, cat. A0564, Dako-Agilent Technologies, Santa Clara, CA, USA) and monoclonal mouse anti-glucagon (dilution 1:300, cat. MAB1249 clone 181402, R&D Systems, Minneapolis, MN, USA) as primary antibodies; subsequently with goat anti-guinea pig Alexa-Fluor 488 conjugate (dilution 1:500, cat. A11073, Molecular Probe, Thermofisher, Waltham, MA, USA), goat anti-rabbit Alexa-Fluor 594 conjugate (dilution 1:500, cat. A11037, Molecular Probe, Thermofisher), goat anti-mouse 647 conjugate (dilution 1:500, cat. A21236, Molecular Probe, Thermofisher) as secondary antibodies. DNA was counterstained with DAPI (4′,6-Diamidino-2-phenylindole dihydrochloride, dilution 1:3,000, cat. D8517, Sigma Aldrich). Sections were mounted with VECTASHIELD (cat. H-1000, Vector Laboratories, Burlingame, CA, USA) antifade medium and analyzed immediately or stored at 4°C until ready for confocal image analysis. Potential non-specific binding of goat anti-mouse-647 conjugate secondary antibody to mouse endogenous immunoglobulins was evaluated through a negative control staining with secondary antibody (without mouse anti-glucagon primary antibody), demonstrating the specificity of the reaction (Supplementary Figure 1).

#### Human Pancreatic Sections

FFPE Pancreatic sections obtained from DiViD and EUnPOD collections were analyzed by triple immunofluorescence using different combinations of antibodies. After deparaffinization and rehydration through alcohol series, 5 μm thick sections were incubated with methanol supplemented with 3% H_2_O_2_ to block endogenous peroxidases and with phosphate-buffered saline (PBS) supplemented with 3% BSA to reduce non-specific reactions. Antigen retrieval was performed with 10 mM citrate buffer pH 6.0 for 10 min. Sections were incubated with polyclonal guinea pig anti-insulin (dilution 1:500, cat. A0564, Dako), monoclonal mouse anti-glucagon (dilution 1:300, cat. MAB1249, clone #181402, R&D Systems), polyclonal rabbit anti-human CXCL10 [dilution 1:300, cat. 500-P93, Peprotech; specific antibody was purified by affinity chromatography employing immobilized hCXCL10 matrix from sera of rabbits pre-immunized with highly pure (>98%) *Escherichia coli* recombinant hCXCL10] (30–32) and/or monoclonal mouse anti-human CD45 (pre-diluted, cat. IR751, clone #2B11 + PD7/26, Dako) as primary antibodies. Subsequently, the following secondary antibodies were adopted: goat anti-guinea pig Alexa-Fluor 488 conjugate (dilution 1:500, cat. A11073, Molecular Probes, Thermofisher), goat anti-mouse Alexa-Fluor 647 conjugate (dilution 1:500, cat. A21236, Molecular Probes, Thermofisher), swine anti-rabbit HRP (dilution 1:100, cat. P0217, Dako), goat anti-guinea pig Alexa-Fluor 647 conjugate (dilution 1:500, cat. A21450, Molecular Probes, Thermofisher), goat anti-mouse Alexa-Fluor 488 conjugate (dilution 1:500, cat. A21236, Molecular Probes, Thermofisher). TSA Fluorescein system (dilution 1:50, cat. NEL742001KT, Perkin Elmer) was used to amplify CXCL10 signal. DNA was counterstained with DAPI. Sections were finally mounted with VECTASHIELD Antifade Medium (Vector Laboratories). Antibodies details and main reagents used in the study are reported in Supplementary Table 1.

### Image Acquisition and Analysis

Images were acquired using Leica TCS SP5 confocal laser scanning microscope system (Leica Microsystems, Wetzlar, Germany). Images were acquired as a single stack focal plane or in *z*-stack mode capturing multiple focal planes (*n* = 5) for each identified islet. Sections were scanned and images acquired at 40 × or 63 × magnification. The same confocal microscope setting parameters (laser power, photomultiplier voltage gain and offset values, pinhole value) were applied to all stained sections before image acquisition in order to uniformly collect detected signal related to each channel.

The analysis of CXCL10, insulin, and glucagon positive signal were performed using Volocity 6.3 software (Perkin Elmer, Waltham, MA, USA). A background threshold filter was uniformly applied to all processed images before the evaluation of specific parameters. A 3D model reconstruction adopting voxels quantification method or pixels, was used to compute single channel signals and to evaluate single channel volumes or the percentage of colocalization coefficient M_1_ (Mander's coefficient) (33) between CXCL10 and insulin and between CXCL10 and glucagon. Colocalization Coefficient M_1_ considers the percentage of pixels (or voxels in case of volume) of a given channel which overlaps to total pixels (or voxels) related to the other channel. Of note, Mander's coefficient is independent of absolute signal as it measures the fraction of one protein that colocalizes with a second protein where *M* represents the fraction (reported in percentage) of colocalizing pixels channel-1/channel-2 on total channel-1 pixels.

Colocalization coefficient M was analyzed for each identified mouse and human islet. In mouse islets, the following parameters were collected for each islet: islet/endocrine volume, insulin volume, glucagon volume, and CXCL10 volume.

### Statistics

Results were expressed as mean ± SD. Statistical analyses were performed using Graph Pad Prism 8 software. Comparisons between two groups were carried out using Mann–Whitney *U* test (for non-parametric data) or Wilcoxon matched signed rank test. Multiple comparisons were analyzed using ordinary one-way ANOVA. Differences were considered significant with *p*-values <0.05.

## Results

### CXCL10 Expression in Pancreas of NOD Mice

CXCL10 expression was not detectable in pancreatic samples from 8-week-old and 20-week-old control C57Bl/6J mice, and absent or barely visible in pancreatic islets of 3-week-old and 20-week-old NOD-SCID mice, which showed no sign of immune cell infiltration or islet inflammation (Supplementary Figure 2).

**Figure 1:**
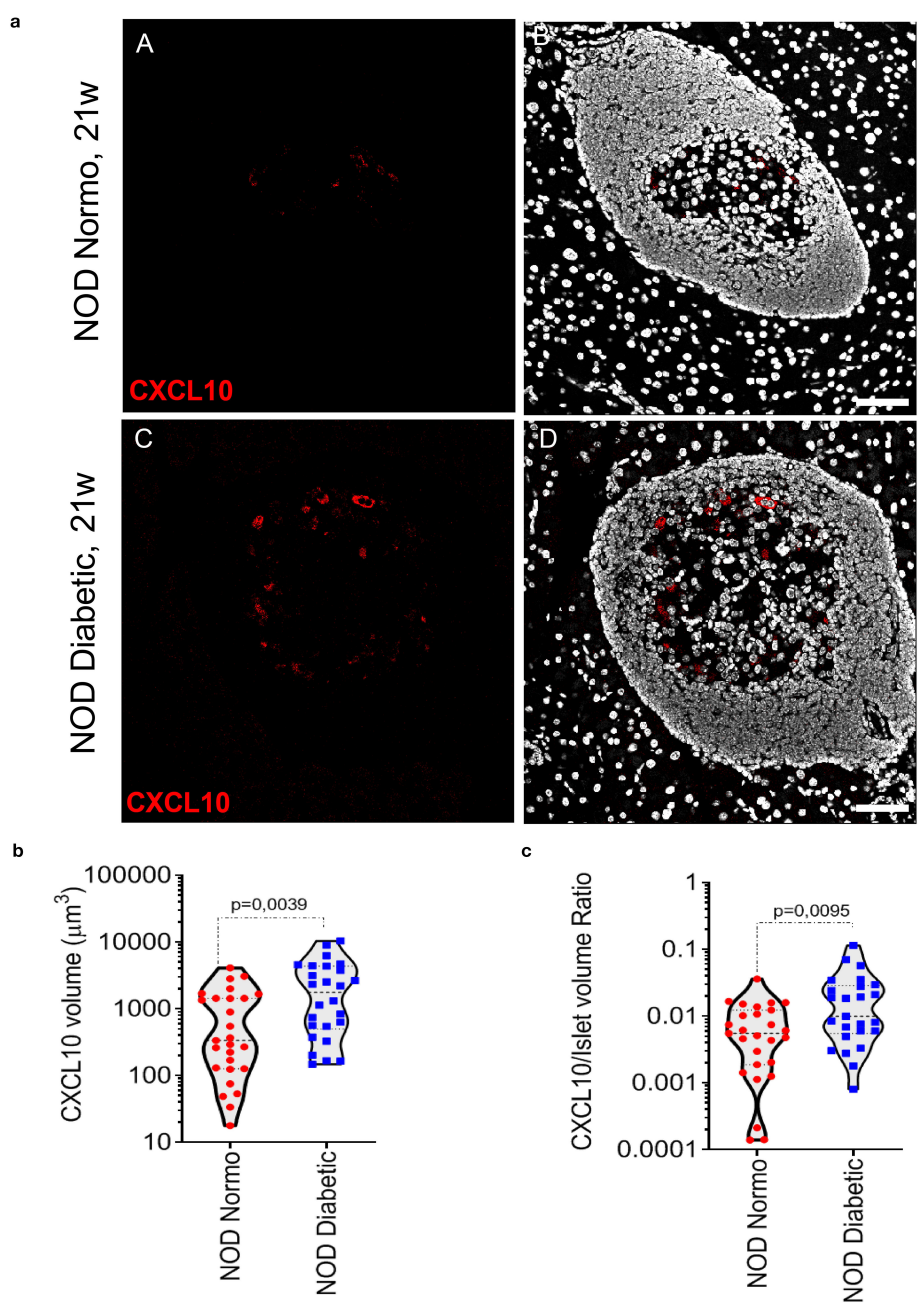
CXCL10 is increased in pancreatic islets of new-onset diabetic NOD mice. **(a)** Immunofluorescence staining of CXCL10 in pancreatic tissue sections of *n* = 4 normoglycaemic NOD mice and in *n* = 4 new-onset diabetic NOD mice. Representative images of normoglycaemic (panels A,B) and new-onset diabetic NOD mice (panels C,D) are reported. CXCL10 is reported in red; nuclei in white/gray. Scale bar is 50 μm. Analysis of CXCL10 total voxels absolute volume **(b)** and normalized per total islet volume **(c)**, in pancreatic islets of *n* = 4 normoglycaemic and *n* = 4 NOD new-onset diabetic NOD mice. A total of *n* = 27 and *n* = 25 pancreatic islets were individually analyzed in normoglycaemic and new-onset diabetic mice, respectively; individual values for each islet are reported in μm^3^ **(b)** or as a volumetric ratio **(c)**. Exact *p*-values were analyzed using non-parametric Mann–Whitney *U* test (*p* < 0.05).

In NOD mice pancreata, CXCL10 expression was not observed in exocrine tissue. In pancreatic islets of 20-22-week-old NOD normoglycaemic mice, CXCL10 expression was weak and localized in few cells within islet parenchyma (Figure 1a, panels A,B). In contrast, in new-onset diabetic NOD mice (12- to 21-week-old) the expression of CXCL10 was higher compared to NOD normoglycaemic mice, as shown by confocal z-stack imaging analysis of pancreatic islets (Figure 1a, panels C,D), revealing an absolute increase of CXCL10 positive volume (Figure 1b), as well as increased CXCL10 signal normalized per total islet volume (Figure 1c). Of note, CXCL10 volume normalization based on beta-cell content, similarly showed an increase of CXCL10 positive signal in pancreatic islets of new-onset diabetic NOD mice (Supplementary Figure 3a). Collectively, these results corroborated previous findings regarding the increase of CXCL10 in pancreatic islets of NOD mice in autoimmune diabetes.

### CXCL10 Is Expressed in Beta- and in Alpha-Cells of NOD Mice

**Figure 2:**
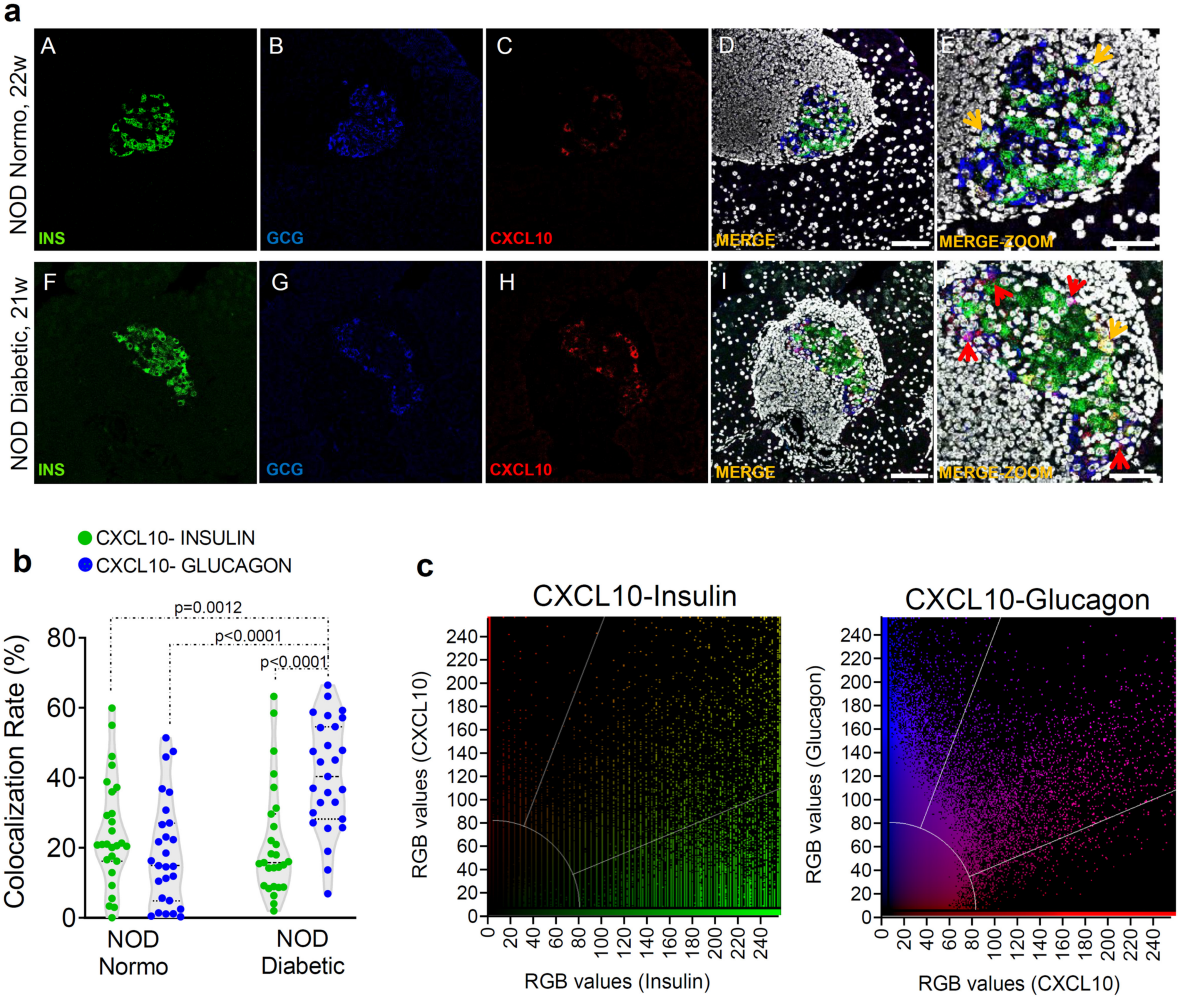
CXCL10 is increased in alpha-cells of new-onset diabetic NOD mice. **(a)** Representative images of triple immunofluorescence reporting the expression of insulin (INS, green), glucagon (GCG, blue), and CXCL10 (red) in pancreatic islets of normoglycaemic (panels A–E) and of new-onset diabetic NOD mice (panels F–J). In panels E,K, zoom-in of pancreatic islets are shown. Colocalization between insulin and CXCL10 is shown in yellow and indicated by yellow arrows; colocalization between glucagon and CXCL10 is reported in magenta and indicated by red arrows. Scale bar in panels D,I = 50 μm. Scale bar panels E,J = 20 μm. **(b)** Colocalization rate are reported as the results of Manders's coefficient evaluation between CXCL10 and insulin (CXCL10-INS) (green dots) and CXCL10 and glucagon (CXCL10-GCG) (blue dots) in individual pancreatic islets of *n* = 4 normoglycaemic NOD mice and *n* = 4 new-onset diabetic NOD mice. Each dot represents an individual islet. A total of *n* = 27 pancreatic islets of normoglycaemic and new-onset diabetic NOD mice are reported. Values are reported as the percentage of CXCL10 signal overlapping with total INS or GCG signal. Exact *p*-values were analyzed using multiple comparison ordinary one-way ANOVA test (*p* < 0.05). Dotted lines represent mean ± SD. **(c)** Colocalization plots of CXCL10-Insulin (left) and CXCL10-glucagon (right) of a new-onset diabetic NOD mouse pancreatic islet. Positive pixels for CXCL10 (red), insulin (green), and glucagon (blue), alongside with colocalizing pixels (CXCL10-insulin: yellow; CXCL10-glucagon: magenta), are reported in the plots. Significant colocalizing pixels are within the area delimited by white lines, representing background and threshold levels relative to each channel. Each pixel is reported as a gray-scale RGB intensity value (0–255).

In order to verify whether the increase of CXCL10 expression observed in islets of new-onset diabetic NOD mice was exclusively dependent on its hyperexpression in beta-cells, we performed triple immunofluorescence staining for CXCL10, insulin and glucagon in pancreas sections of normoglycaemic and new-onset diabetic NOD mice. Interestingly, CXCL10 was expressed both in beta- and in alpha-cells (Figure 2a), suggesting that its expression is not an exclusive feature of beta-cells. Of note, in NOD normoglycaemic mice, colocalization analysis between CXCL10-insulin and CXCL10-glucagon revealed that the proportion of beta- and alpha-cells positive for CXCL10 was similar [CXCL10-INS 24.3 ± 15.3% vs. CXCL10-GCG 18.7 ± 15.2% (mean ± SD), respectively] (Figure 2b). In new-onset diabetic NOD mice, CXCL10-glucagon colocalization was significantly higher compared to CXCL10-insulin [40.6 ± 15.7% vs. 21.3 ± 16.0% (mean ± SD) *p* < 0.0001] (Figures 2b,c). Moreover, CXCL10-insulin colocalization did not differ between normoglycaemic and new-onset diabetic NOD mice [24.3 ± 15.3% vs. 21.3 ± 16.0% (mean ± SD)], suggesting that alpha-cells significantly contribute to the increase of CXCL10 in pancreatic islets of new-onset diabetic NOD mice. Importantly, such increase was not dependent on changes in islets volume (Supplementary Figure 3b).

### Four Different Islet Subsets Can Be Identified in Pancreata of New-Onset T1D Patients Based on CXCL10 Expression Pattern

Results obtained in NOD mice demonstrated a peculiar CXCL10 expression pattern, pointing out to a significant involvement of alpha-cells in chemokine secretion in T1D. These findings prompted us to investigate its distribution in pancreatic islets of T1D donors.

Firstly, we further confirmed that CXCL10 was absent in pancreatic islets of non-diabetic donors. We analyzed pancreatic sections derived from 3 EUnPOD-INNODIA non-diabetic multiorgan donor (subjects characteristics reported in Table 1) using triple immunofluorescence analysis for CXCL10, insulin and glucagon. Results demonstrated that CXCL10 was not expressed in non-diabetic pancreatic islets (Supplementary Figure 4). Then, we analyzed CXCL10 expression pattern distribution in pancreatic sections of 6 new-onset (≤9 weeks from diagnosis) T1D subjects from DiViD study (Table 1).

**Figure 3:**
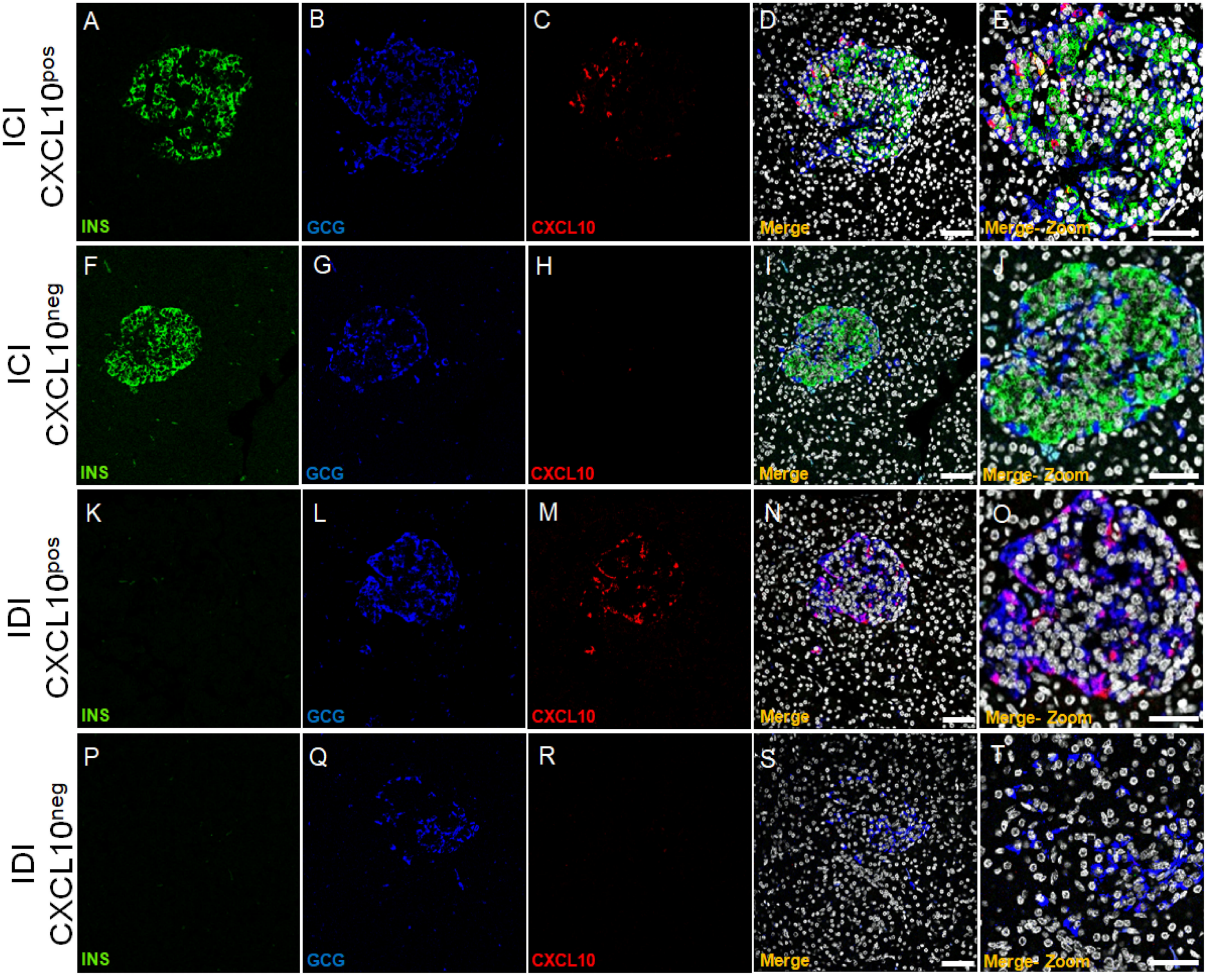
CXCL10 is expressed in ICIs and IDIs of new-onset diabetic individuals and distinguished four pancreatic islet subsets. Triple immunofluorescence analysis of insulin (INS, green), glucagon (GCG, blue), and CXCL10 (red) in pancreatic sections of new-onset T1D DiViD cases. Representative pancreatic islet 40 × confocal microscope images are shown for each channel, alongside with digital zoom-in for each set of panel. **(A–E)** ICI showing positivity for CXCL10. **(F–J)** ICI without CXCL10 positivity. **(K–O)** IDI showing CXCL10 positivity. **(P–T)** IDI without positivity for CXCL10. Scale bar =100 μm. Scale bar zoom-in = 40 μm.

CXCL10 expression was not observed in exocrine tissue nor in CD45^+^ cells surrounding or infiltrating pancreatic islets or scattered in acinar tissue (Supplementary Figure 5). In line with previous reports, we confirmed that CXCL10 was expressed in pancreatic islets but not in exocrine/acinar tissue. Of note, a heterogeneous pattern of CXCL10 expression among pancreatic islets of T1D DiViD cases was clearly observed. Indeed, based on CXCL10 positivity and on the presence or absence of insulin [insulin-containing islets (ICIs) and insulin-deficient islets (IDIs)], four different islet subsets can be readily distinguished: ICIs with CXCL10 expression (ICI-CXCL10^pos^) and ICIs without any sign of CXCL10 positivity (ICI-CXCL10^neg^); IDIs containing CXCL10 positive cells (IDI-CXCL10^pos^) and IDIs without any positivity for the chemokine (IDI-CXCL10^neg^) (Figure 3).

For each T1D DiViD case, we analyzed two non-consecutive sections derived from two different paraffin blocks of the pancreas tail. Overall, using fluorescent confocal microscopy, we manually screened a total of 1,148 pancreatic islets from 6 new-onset T1D DiViD subjects. Of 1,148 islets, 343 were ICIs and 805 were IDIs, in line to what has been previously observed from multiple histological analyses of the same DiViD cases, showing a higher number of IDIs vs. ICIs. Of 343 ICIs, the majority (*n* = 332, 96.7% of the total ICIs) were positive for CXCL10, while 11 (3.3%) were negative for the chemokine. Interestingly, among 805 IDIs, 514 (63.8%) contained CXCL10 positive cells, while 291 (36.1%) were negative (Supplementary File 1).

**Figure 4:**
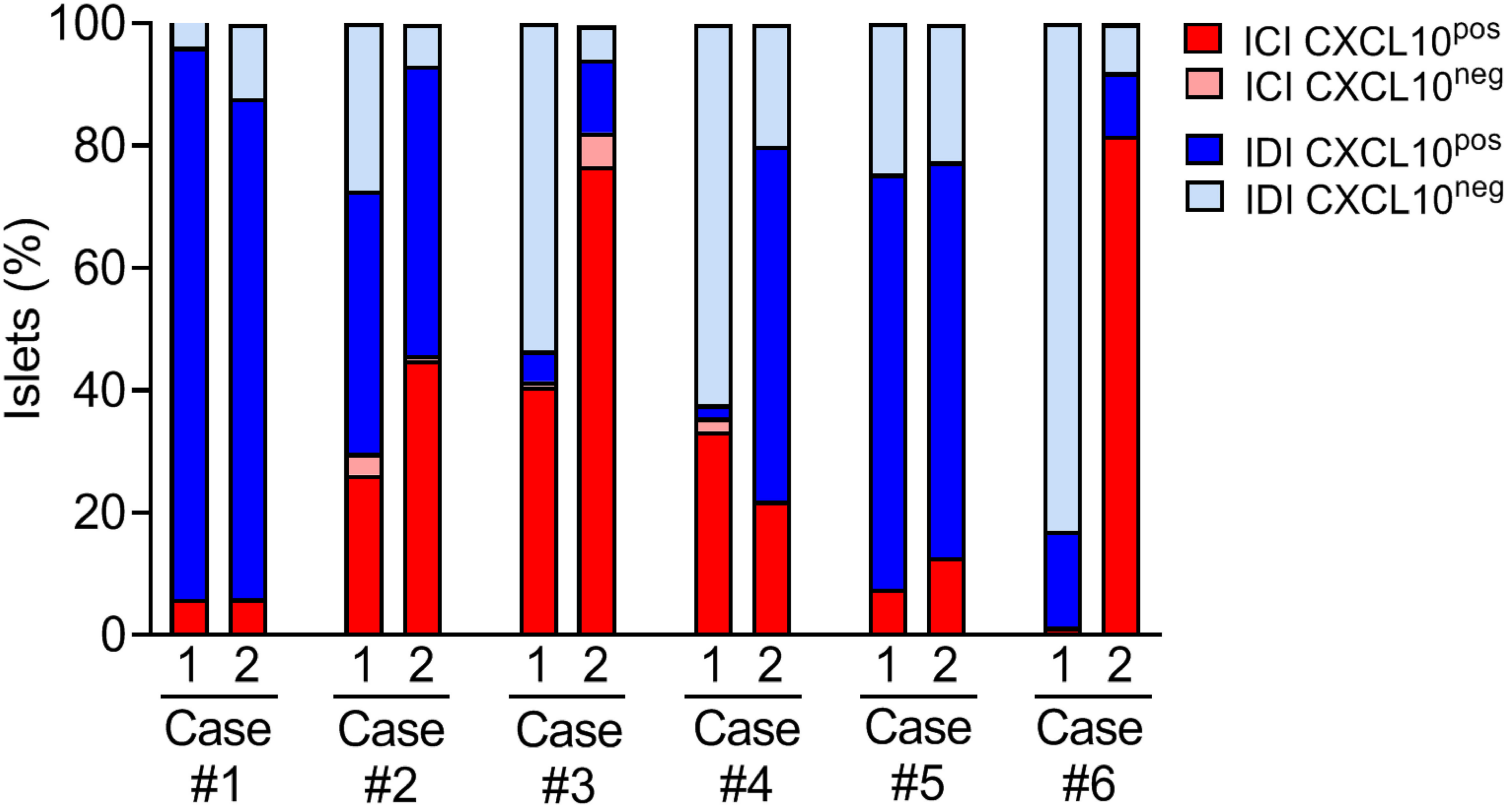
Distribution of islet subsets in T1D DiViD individuals based on CXCL10 expression and ICI/IDI classification. Histological evaluation of islet subsets distribution in each of the six recent-onset DiViD individuals, based on the analysis of two non-consecutive pancreatic sections/case. Distribution of islets is reported as percentage value of total islets identified per section.

A case-by-case analysis showed a heterogeneous CXCL10 staining pattern and distribution of islet subsets, confirming a substantial heterogeneity among T1D individuals (Figure 4 and Table 2). In all cases, regardless of the section analyzed, almost all ICIs were positive for CXCL10.

**Table 2:** Table reporting the percentage values and the absolute number (in parentheses) of ICIs and IDIs positive or negative for CXCL10 in two non-consecutive pancreatic sections derived from two different formalin-fixed paraffin-embedded pancreatic tissue histological blocks of the same DiViD case.

	**Section #**	**ICI CXCL10^POS^ % (absolute)**	**ICI CXCL10^NEG^ % (absolute)**	**IDI CXCL10^POS^ % (absolute)**	**IDI CXCL10^NEG^ % (absolute)**
Case 1	Section #1	5.9 (3)	0 (0)	90.2 (46)	3.9 (2)
	Section #2	6.0 (5)	0 (0)	81.7 (67)	12.2 (10)
Case 2	Section #1	26.2 (22)	3.5 (3)	42.9 (36)	27.4 (23)
	Section #2	44.9 (53)	0.8 (1)	47.4 (56)	6.77 (8)
Case 3	Section #1	40.6 (41)	0.9 (1)	5 (5)	53.5 (54)
	Section #2	76.6 (69)	5.5 (5)	12.0 (11)	5.5 (5)
Case 4	Section #1	33.3 (15)	2.2 (1)	2.2 (1)	62.2 (28)
	Section #2	21.9 (23)	0 (0)	58.0 (61)	20 (21)
Case 5	Section #1	7.6 (16)	0 (0)	67.8 (143)	24.6 (52)
	Section #2	12.7(13)	0 (0)	64.7 (66)	22.5 (23)
Case 6	Section #1	1.4 (1)	0 (0)	15.7 (11)	82.9 (58)
	Section #2	81.6 (71)	0 (0)	10.3 (9)	8.0 (7)

*See Supplementary File 1 for an extended version of this table*.

In both sections analyzed, Case-1, Case-2, and Case-5 showed a consistent and significant higher proportion of IDI-CXCL10^pos^ compared to IDI-CXCL10^neg^ (Figure 4). Of note, in these cases, IDIs represent the major source of CXCL10, being higher compared to ICI-CXCL10^pos^ (Figure 4, Table 2, and Supplementary File 1).

In contrast, in Case-3, Case-4, and Case-6, we found a striking heterogeneity between the two sections analyzed, mainly due to the different rate of IDIs positive for CXCL10 (Figure 4). Notable, in Case-6, the high heterogeneity observed in terms of ICIs and IDIs presence (section#1: 1.5% ICIs vs. 98.5% IDIs; section#2: 81.6% ICIs vs. 18.4% IDIs) between the two sections is paralleled by strong differences in CXCL10 islets positivity (Figure 4, Table 2, and Supplementary File 1) being more frequent in section#2 within ICIs (100% of ICIs CXCL10^pos^) compared to IDIs in section#1 (15.7% of IDIs CXCL10^pos^).

### Alpha-Cells Contribute to CXCL10 Expression in Pancreatic Islets of New-Onset T1D Patients

**Figure 5:**
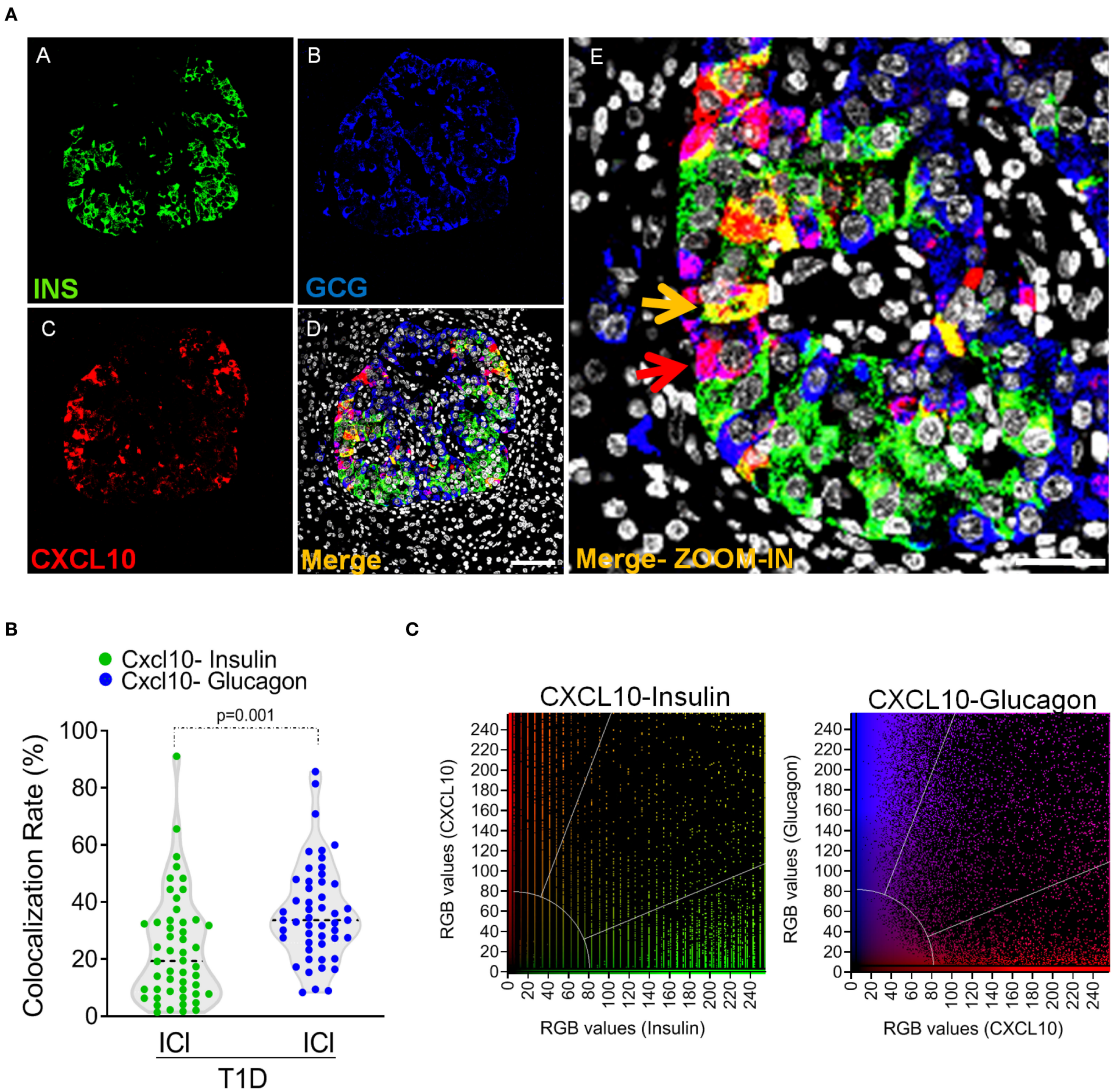
Alpha-cells contribute to CXCL10 expression in islets of new-onset T1D patients. **(a)** Triple immunofluorescence analysis of insulin (INS, green, panel A), glucagon (GCG, blue, panel B), and CXCL10 (red, panel C) of pancreatic islets in T1D DiViD cases. Panel E: digital zoom-in of overlapping (merge) channels, showing colocalization of CXCL10 and insulin (yellow pixels) indicated by yellow arrow and of CXCL10 and glucagon (magenta pixels) indicated by red arrow. Scale bar = 50 μm. Scale bar zoom-in = 20 μm. **(b)** Colocalization analysis of CXCL10 and insulin (green dots) and CXCL10 and glucagon (blue dots) in pancreatic islets of T1D DiViD cases. A total of *n* = 50 ICIs from 6 DiViD cases were analyzed for both CXCL10-insulin and CXCL10-glucagon colocalization rate. Values are reported as the percentage of overlapping CXCL10-insulin or CXCL10-glucagon pixels over total insulin or glucagon positive pixels, according to Mander's Coefficient calculation. Exact *p*-value was calculated using Wilcoxon matched-pairs signed rank test. **(c)** Colocalization plots of CXCL10-insulin (left) and CXCL10-glucagon (right) of a recent-onset diabetic DiViD individual ICI (Case-1). Positive pixels for CXCL10 (red), insulin (green), and glucagon (blue), alongside with colocalizing pixels (CXCL10-insulin: yellow; CXCL10-glucagon: magenta), are reported in the plots. Significant colocalizing pixels are within the area delimited by white lines, representing background and threshold levels relative to each channel. Each pixel is reported as a gray-scale RGB intensity value (0–255).

The relevant presence of IDIs showing positivity for CXCL10 strongly suggests that also in human context, CXCL10 expression is not exclusively expressed by beta-cells. Indeed, triple immunofluorescence staining aimed at detecting insulin, glucagon, and CXCL10 expression in pancreatic sections of 6 new-onset T1D subjects from DiViD study, demonstrated that: (a) in ICIs, both beta- and alpha-cells stained positive for CXCL10 (Figure 5a and Supplementary Figure 6); (b) in IDIs, CXCL10 was expressed only in alpha-cells, since the (whole) signal of the chemokine perfectly overlapped with glucagon (Figures 3K–O and Supplementary Figure 7).

In order to quantify the contribution of beta- and alpha-cells to the overall expression of CXCL10 in pancreatic islets of T1D subjects, we analyzed the colocalization rate of CXCL10-insulin and CXCL10-glucagon in ICIs detected in all DiViD cases. Such analysis demonstrated that CXCL10-glucagon colocalization rate was significantly higher compared to CXCL10-insulin [CXCL10-GCG 36.5 ± 17.1% vs. CXCL10-INS 23.6 ± 18.9% (mean ± SD) (Figures 5b,c)], thus demonstrating that alpha-cells significantly contribute, together with beta-cells, to CXCL10 expression in pancreatic islets of T1D subjects.

## Discussion

Several studies reported that CXCL10 expression is increased in *in-vitro* cultured pancreatic islets upon inflammatory stresses (34, 35), as well as in pancreatic islets of animal models of autoimmune diabetes (6, 7) and in donors with T1D (24–26). However, data are lacking regarding CXCL10 intra-islet expression pattern in T1D. Such context prompted us to further investigate CXCL10 expression in pancreas sections of NOD mice and of T1D subjects from DiViD study, in order to better define CXCL10 intra-islet distribution.

In the present study, we confirmed that CXCL10 was expressed in pancreatic islets but not in exocrine tissue in T1D, while its expression was not observed in pancreas of healthy donors. Our data are in line with previous reports showing increased expression of CXCL10 in pancreatic islets in T1D (23–25).

Interestingly, our data suggest that both beta- and alpha-cells contribute to CXCL10 expression in T1D pancreatic islets, both in diabetic NOD mice and in DiViD T1D subjects.

In 12- to 21-week-old new-onset diabetic NOD mice, CXCL10 was expressed in pancreatic islets, but not in exocrine tissue, and significantly increased vs. age-matched normoglycaemic NOD mice.

Our results show a significant increase in the proportion of alpha-cells expressing CXCL10 in new-onset diabetic vs. normoglycaemic NOD mice, thus potentially suggesting that a higher rate of alpha-cells are subjected to inflammatory stresses and respond by activating CXCL10 transcription.

These findings are mirrored in pancreata of T1D DiViD subjects compared to healthy multiorgan donors collected within the EUnPOD network of INNODIA consortium. In line with previous studies (24–26), we confirmed that CXCL10 was specifically expressed in pancreatic islets of T1D subjects and absent in non-diabetic controls. In ICIs, CXCL10 expression was observed both in beta- and in alpha-cells. As expected, in all DiViD cases analyzed, most of the ICIs (95%) showed positivity for CXCL10, in line with previous observations which attributed a more aggressive insulitis and inflammation to those islets containing residual beta-cells (36). Of interest, in ICIs we observed a higher proportion of CXCL10 positive alpha-cells compared to beta-cells, suggesting a critical contribution of alpha-cells to the pancreatic islet expression of CXCL10. To this regard, it should be underlined that Mander's colocalization coefficient is independent of absolute signal as it measures the fraction of one protein that colocalizes with a second protein; therefore, it is unlikely that the differences observed in the colocalization rates are dependent on beta- or alpha-cell mass modifications.

Strikingly, the expression of CXCL10 was also clearly observed in alpha-cells of IDIs where beta-cells were absent and inflammation was lower or not present, as shown previously (37–40) and in the present manuscript as well (Supplementary Figures 4a,b). Based on manual counting of IDI-CXCL10^pos^ in each DiViD case, we observed that Case-1, Case-2, and Case-5 revealed a higher fraction of IDI-CXCL10^pos^ among all IDIs detected; this result is consistent between the two non-consecutive pancreatic sections analyzed. Conversely, a substantial heterogeneity between the two sections was observed in Case-3, Case-4, and Case-6, mainly due to the different rate of ICI-CXCL10^pos^, clearly evident in Case-6. Despite the high heterogeneity, overall, Case-3, Case-4, and Case-6 showed the lowest proportion of IDI-CXCL10^pos^ (Supplementary File 1). In an effort aimed at looking for specific characteristics correlated with CXCL10-based DiViD cases patterning, we found that Case-6, showing the lowest rate of CXCL10^pos^ islets (considering both sections and independently of its cellular distribution) (Supplementary File 1), also exhibited the lowest expression of *HLA-ABC* genes among DiViD cases, as previously reported by Richardson S and colleagues (38). Additionally, in Case-3, classified by having high residual beta-cell content, severe insulitis and high expression of HLA Class-I (37, 38), we observed the highest proportion of ICI-CXCL10^pos^ among all DiViD cases.

Collectively, these results suggest that, although residual beta-cells drive severe pancreatic islet inflammation leading to a global CXCL10 increase, the expression of this chemokine in alpha cells could represent a phenomenon not strictly dependent on beta-cell content. Of note, a very high level of heterogeneity was observed among cases analyzed and among different paraffin blocks of the same case, in line with the heterogeneous nature of the disease, previously highlighted by several studies assaying the same cases (28, 37, 38, 41).

In support of our data, CXCL10 hyperexpression in DiViD cases was also previously observed at the mRNA level, being its expression significantly increased in laser-captured microdissected islets of T1D donors compared to non-diabetic controls (42); of note, CXCL10 hyperexpression was reported to be significantly associated to peri-islet insulitis microdissected tissue rather than to pancreatic islets core. Such results are in line with our data; indeed, it is likely that CXCL10 hyperexpression observed in peri-islet/insulitic microdissected tissue from T1D donors was mostly derived from alpha-cells clusters which are more closely associated to the peri-islets basement membrane (43). In addition, our results exclude an overlapping between insulitic immune cells and CXCL10 expression as shown by CD45-CXCL10 immunofluorescence staining in T1D DiViD sections (Supplementary Figure 4a).

In support to our findings, CXCL10 expression in alpha-cells was previously reported by Tanaka et al. in Japanese fulminant diabetes cases (26) and, more recently, by Moin et al. (44) in pancreatic islets of multiorgan donors with chronic pancreatitis, thus confirming and extending the observation of CXCL10 expression in alpha-cells in autoimmune diabetes.

Of interest, our data corroborate the increasing importance attributed to alpha-cells in the pathogenesis and progression of T1D. Alterations of several genes alongside with functional defects have been observed in alpha-cells obtained from T1D donors. These include alterations of alpha-cells phenotypic-maintenance genes and defects in glucagon secretion (45). We can speculate that inflammation may contribute to the activation of several signaling pathways, which alter alpha-cells phenotype and activate innate inflammatory responses leading to CXCL10 expression. As a matter of fact, CXCL10 is not the only pro-inflammatory molecule expressed by alpha-cells; indeed, it has been reported that alpha-cells can express also IL-1β (46) as well as IL-6 (47), thus potentially contributing to the pro-inflammatory islet microenvironment causing preferential homing of T-lymphocytes in pancreas in T1D (48). In turn, increased immune cell migration and then inflammation could enhance beta-cell antigenicity through higher HLA Class-I expression and novel peptides exposure to the immune system (49), thus generating a critical positive feedback loop.

An additional layer of evidence, supporting the expression of CXCL10 by alpha-cells, is given by their molecular equipment needed to induce those signaling pathways which lead to CXCL10 transcriptional activation. Indeed, analysis of transcriptome datasets comparing beta- and alpha-cells gene expression, showed an almost equal expression levels of those receptors and intracellular molecules which initiate the signaling cascades leading to CXCL10 transcriptional activation, such as IFNAR1, IFNAR2, IFNGR, TYK2, TNFRSF1A, and IL-1R (50–53), thus demonstrating the potential ability of alpha-cells to respond to the inflammatory milieu and potentially activate CXCL10 pathway.

However, several open questions remain. Firstly, the potential role of CXCL10 beside its effects on immune cells recruitment needs to be clarified; several reports attributed a role for CXCL10 in proliferation and angiogenesis (54). Particularly, it has been reported that CXCL10 can modulate vascular angiogenesis (55), also through the inhibition of VEGF-A (56). Angiogenesis has been linked to beta-cell regeneration through the re-arrangement of islet microenvironment, thus hypothesizing a role for islet CXCL10 as a factor involved in the modulation of beta-cell regeneration (57).

Secondly, the presence of CXCL10 in IDIs with no sign of inflammation may suggest that CXCL10 transcriptional activation is not only induced by cytokines and inflammatory mediators but may be caused by the exposure to additional factors. In this regard, alternative signaling pathways and receptors (e.g., TLR4) have been reported for the induction of CXCL10 (58).

Thirdly, the co-existence of IDI-CXCL10^pos^ and IDI-CXCL10^neg^ indicates a high level of heterogeneity involving also pancreatic islets alpha-cells expressing CXCL10; the identification of those factors determining the expression of CXCL10 in alpha-cells and how these correlate with individual islet phenotype would be of major importance to understand the role of this chemokine in T1D.

In conclusion, we have shown that chemokine CXCL10 is expressed also by alpha-cells which represent important contributors to the expression of CXCL10 in pancreatic islets. These results further underline the role of alpha-cells in T1D pathogenesis and progression and suggest the need to advance our knowledge regarding function and dysfunction of these cells in pancreatic islet autoimmunity.

## Data Availability Statement

All datasets generated for this study are included in the article/Supplementary Material.

## Ethics Statement

The studies involving human participants were reviewed and approved by Norwegian Governments Regional Ethics Committee. Written informed consent was obtained from all individuals with type 1 diabetes after they had received oral and written information from the diabetologist and the surgeon separately. EUnPOD multiorgan donors' pancreata not suitable for clinical purposes were obtained with informed written consent by organ donors' next-of-kin and processed with the approval of the local ethics committee of the Pisa University. Specific consent to the publication of the data was obtained from the participants or by organ donors' next-of-kin. The patients/participants provided their written informed consent to participate in this study. The animal study was reviewed and approved by Ethics Committee of the KU Leuven. All animal procedures were performed in accordance with the NIH guidelines for the care and use of laboratory animals. Written informed consent was obtained from the individual(s) for the publication of any potentially identifiable images or data included in this article.

## Author Contributions

LN, NB, and GS performed the experiments, analyzed the data, and wrote the manuscript. GG analyzed the data and contributed to the scientific discussion. GL performed the experiments during the revision stage and contributed to the scientific discussion. LN, GS, and FD reviewed the manuscript and designed experiments. LK and KD provided support for DiViD cohort and contributed to the scientific discussion. CG and LO reviewed the manuscript, provided support for animal models, and contributed to the scientific discussion. CM reviewed the manuscript and contributed to the scientific discussion. LM and PM reviewed the manuscript, provided support for EUnPOD donors, and contributed to the scientific discussion. All authors contributed to the article and approved the submitted version.

## Conflict of Interest

The authors declare that the research was conducted in the absence of any commercial or financial relationships that could be construed as a potential conflict of interest. The handling Editor declared a past co-authorship with the authors LN, LM, FD, GS, KD, PM, CG, LO, and CM.
