# Supplementary material for: Pancreatic Alpha-Cells Contribute Together With Beta-Cells to CXCL10 Expression in Type 1 Diabetes
Source: Front Endocrinol (Lausanne). 2020 Sep 15;11:630. doi: 10.3389/fendo.2020.00630 (PMC7523508; doi:10.3389/fendo.2020.00630)
Supplement: Supplementary file 2 [file Data_Sheet_1.PDF]

Supplementary Material

1.1 Supplementary Figures

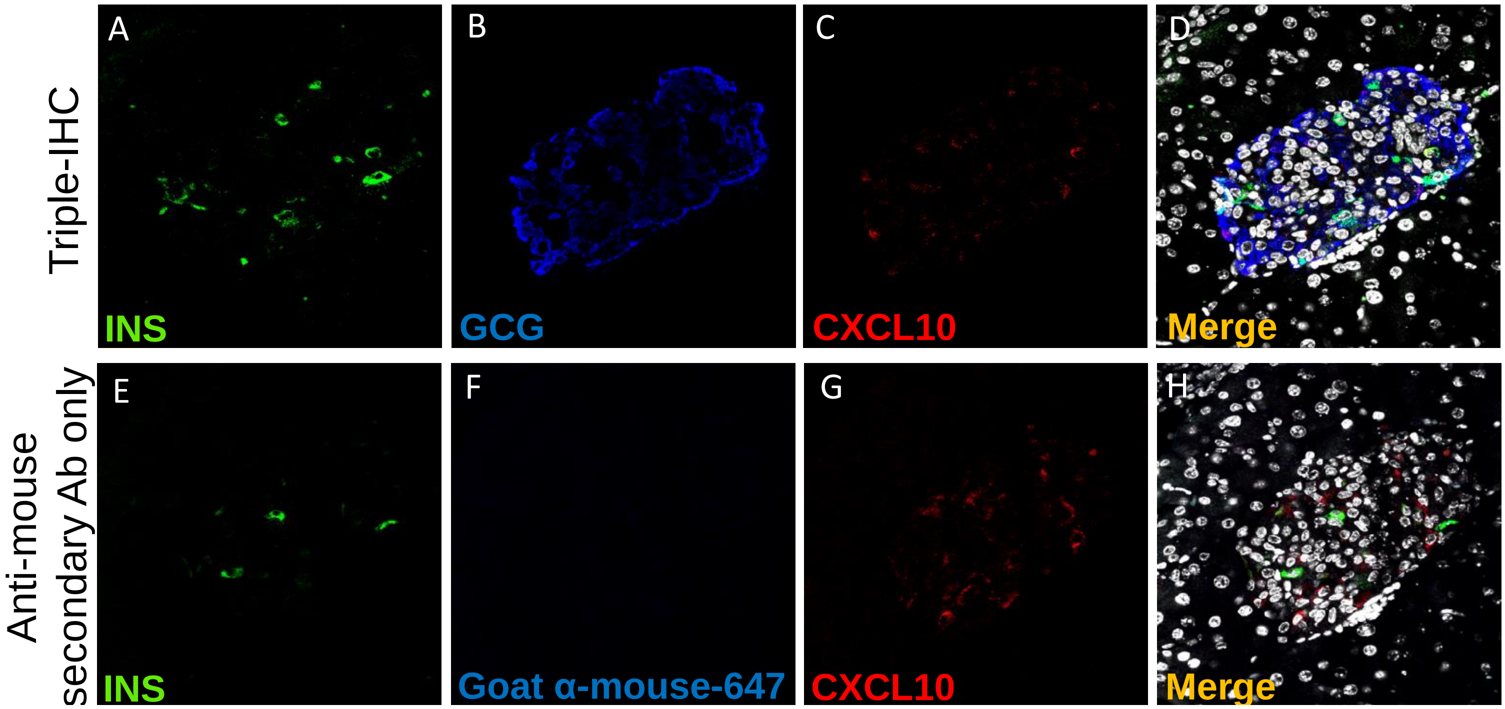

Supplementary Figure 1. **Mouse anti-Glucagon negative control staining**. Panel-A to -D: triple immunofluorescence staining in a NOD recent onset diabetic pancreatic section reporting the expression of insulin (green), glucagon (blue) and CXCL10 (red). Panel-E to -H: immunofluorescence staining in the serial section of that reported in panel-A to D, stained without the primary antibody mouse-anti-glucagon (only secondary Ab negative control). The staining demonstrates the specificity of the mouse-on-mouse staining for the detection of glucagon without artifacts or background noise.

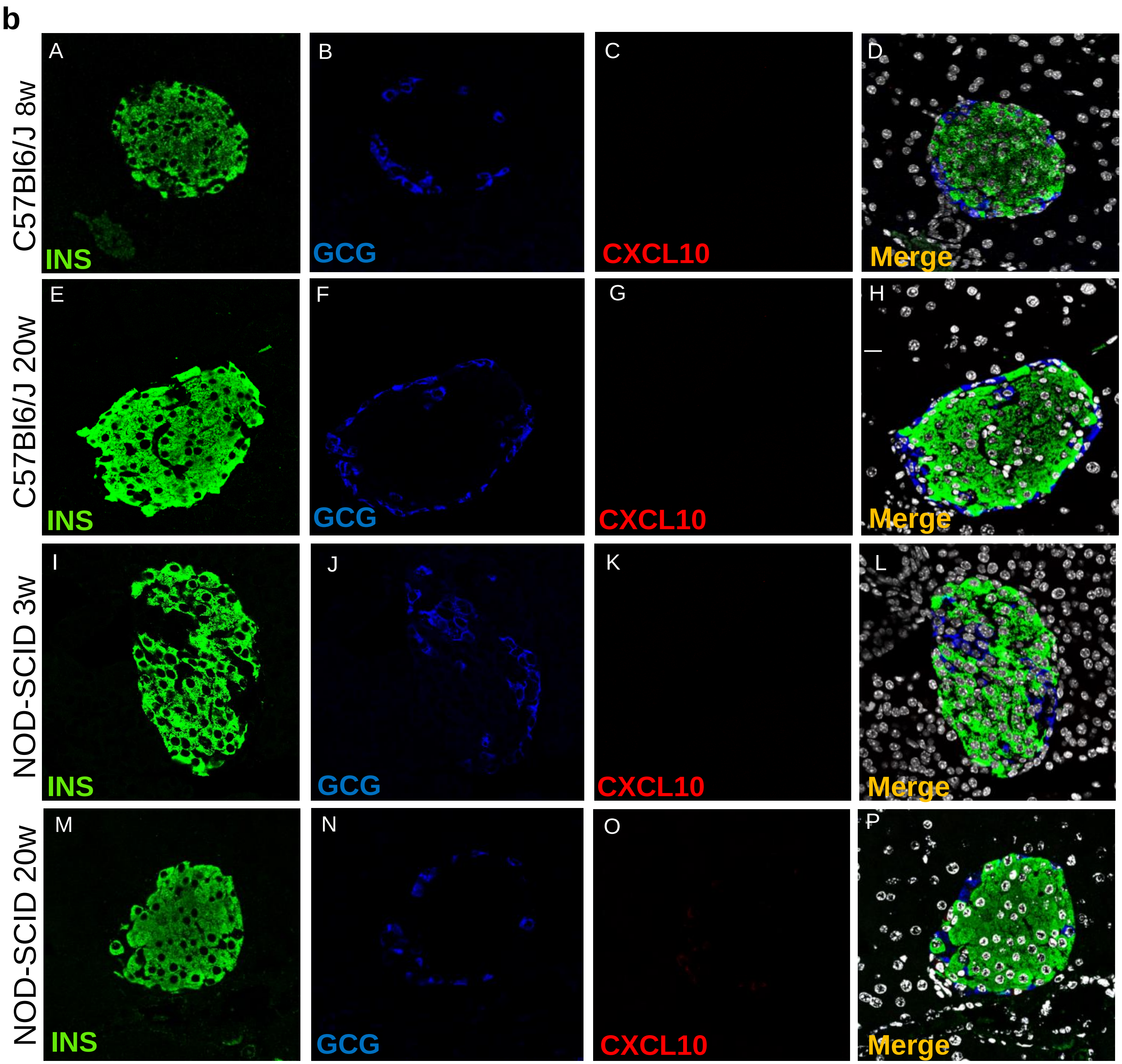

Supplementary Figure 2. **CXCL10 is not expressed in pancreatic islets of C57Bl6/J and NOD-SCID mice**. Representative images of pancreatic tissue sections reporting the expression of insulin (INS, green), glucagon (GCG, blue) and CXCL10 (red) in pancreatic islets of C57Bl6/J 8 week, 20

week and of NOD-SCID 3 week and 20 week mice. Panel-A to -D: C57Bl6/J 8 week. Panel-E to -H: C57Bl6/J 20 week. Panel-I to -L: NOD-SCID 3 week. Panel-M to -P: NOD-SCID 20W.

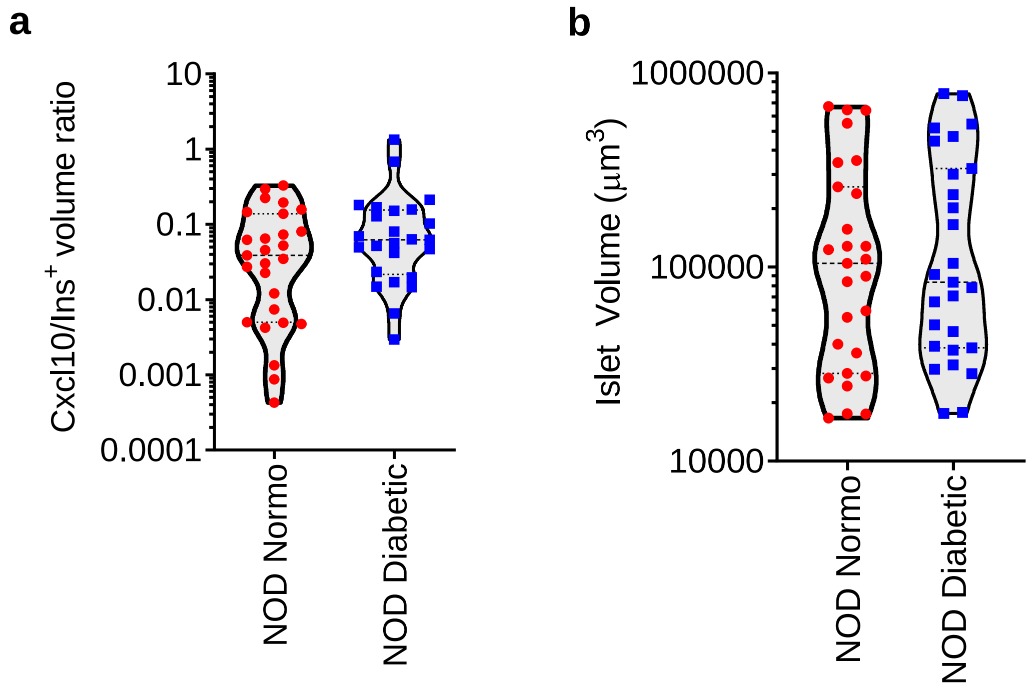

Supplementary Figure 3. (a) Analysis of CXCL10/insulin positive volume ratio showing increased levels of CXCL10 in new-onset diabetic NOD mice. A total of n=27 and n=25 pancreatic islets were individually analyzed in normoglycemic and new-onset diabetic NOD mice, respectively; individual values for each islet are reported as a ratio between CXCL10 and insulin. (**b**) Plot reporting islets volume in normoglycaemic and new-onset diabetic NOD mice for CXCL10 normalization analysis. Values are reported as μm^3.

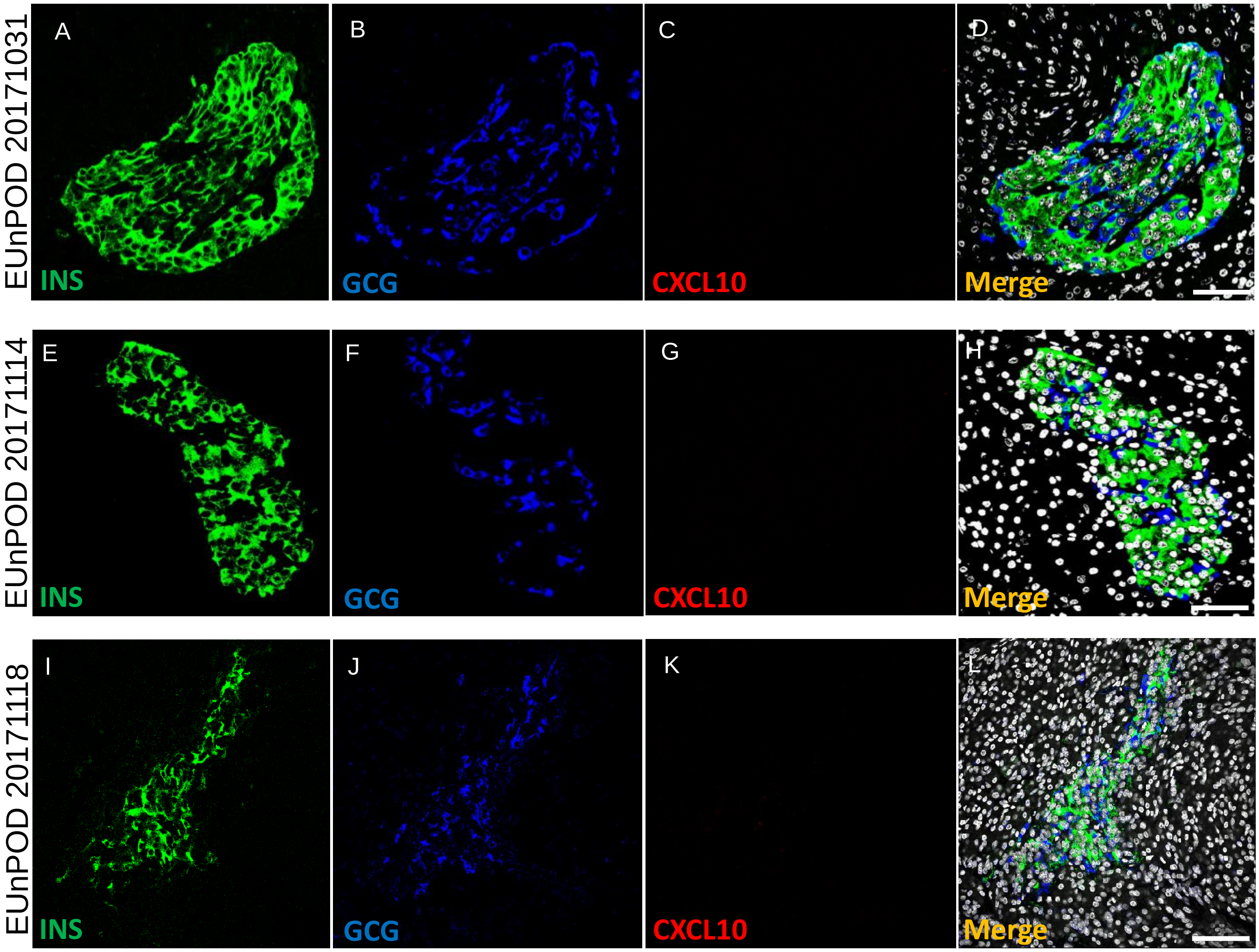

Supplementary Figure 4. CXCL10 is not expressed in pancreatic islets of non-diabetic multiorgan donors._Pancreatic tissue sections triple immunofluorescence representative images reporting the expression of insulin (INS, green), glucagon (GCG, blue) and CXCL10 (red) in pancreatic islets of EUnPOD donors. Scale bars: panel-D and H=100 µm; panel-L=150 µm.

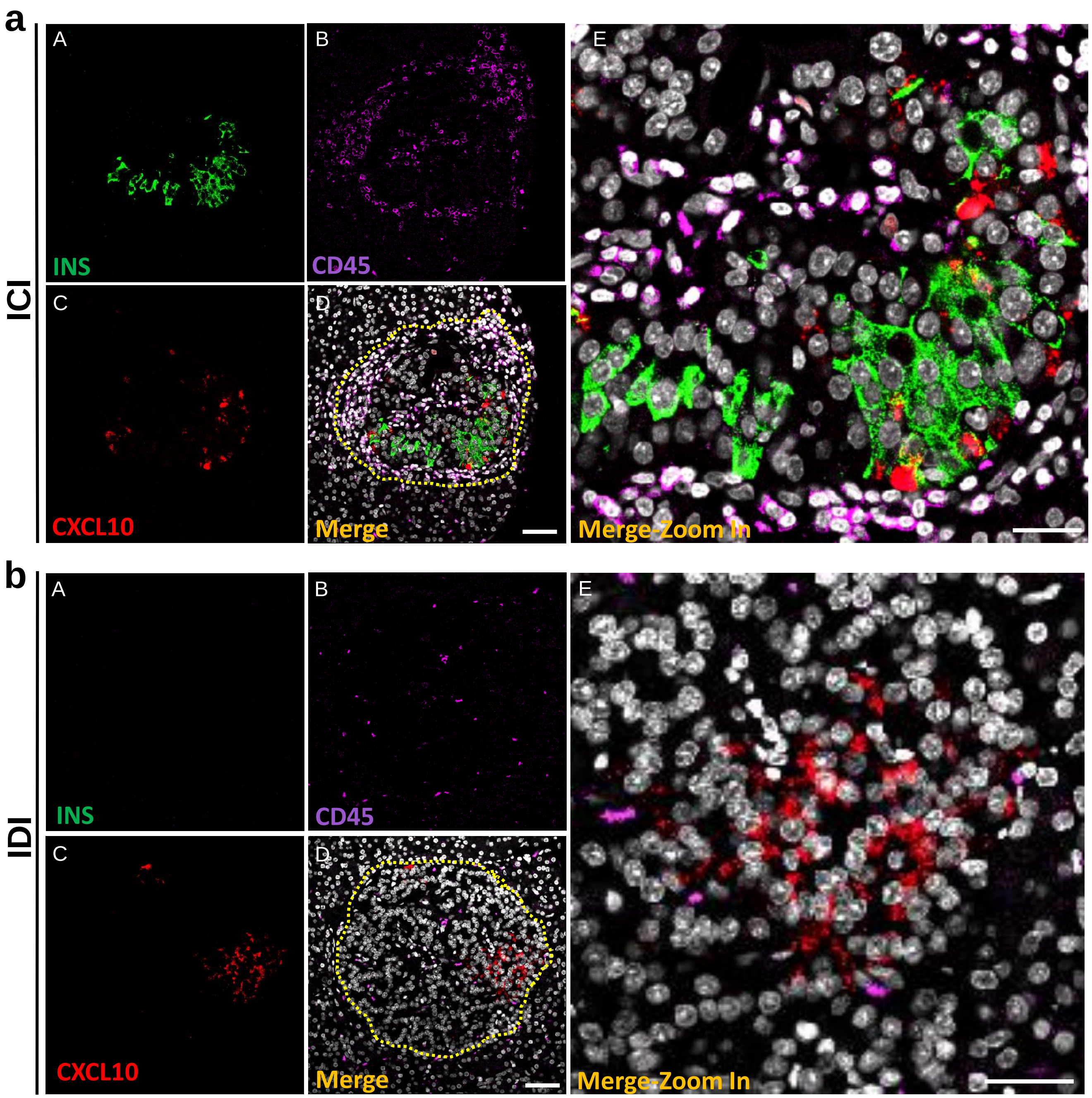

Supplementary Figure 5. **CD45-CXCL10-INS triple IHC immunofluorescence in T1D pancreatic sections**. (**a**) Representative image of a triple immunofluorescence staining in a pancreatic section from T1D DiViD case-3, showing a CXCL10-positive insulin containing islet (ICI) surrounded by CD45$^+$ immune cells [insulin (INS, green), CD45 (magenta) and CXCL10 (red)]. Scale Bar panel-D=75µm; scale bar panel-E=20µm. (**b**) Representative image of CD45-CXCL10-INS staining of a pancreatic

section from T1D DiViD case-5, showing a CXCL10-positive insulin-deficient islet (IDI), without any surrounding or infiltrating CD45^{+} immune cells [insulin (INS, green), CD45 (magenta) and CXCL10 (red)]. Scale Bar panel-D=75µm; scale bar panel-E=20µm.

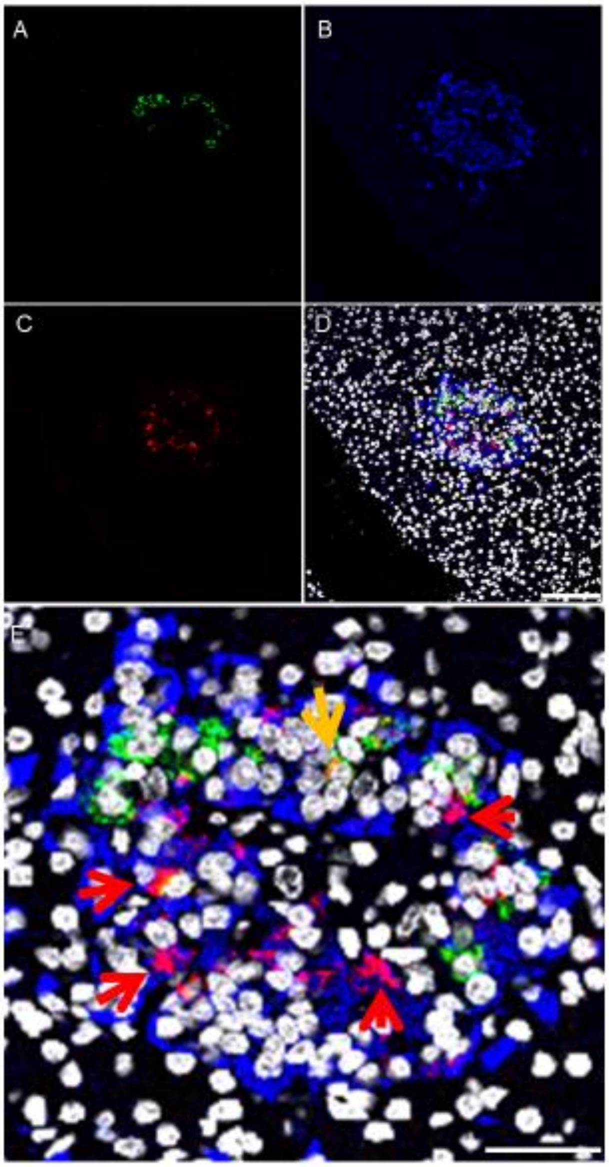

Supplementary Figure 6. CXCL10 expression distribution in ICI. Example of an ICI positive for CXCL10 showing prevalent expression of CXCL10 in alpha-cells compared to beta-cells. Panel A: insulin (green); panel-B: glucagon (blue); panel-C: CXCL10 (red); panel-D (merge). Nuclei (DAPI): white/grey. Scale bar panel-D: 100 µm; panel-E: 35 µm.

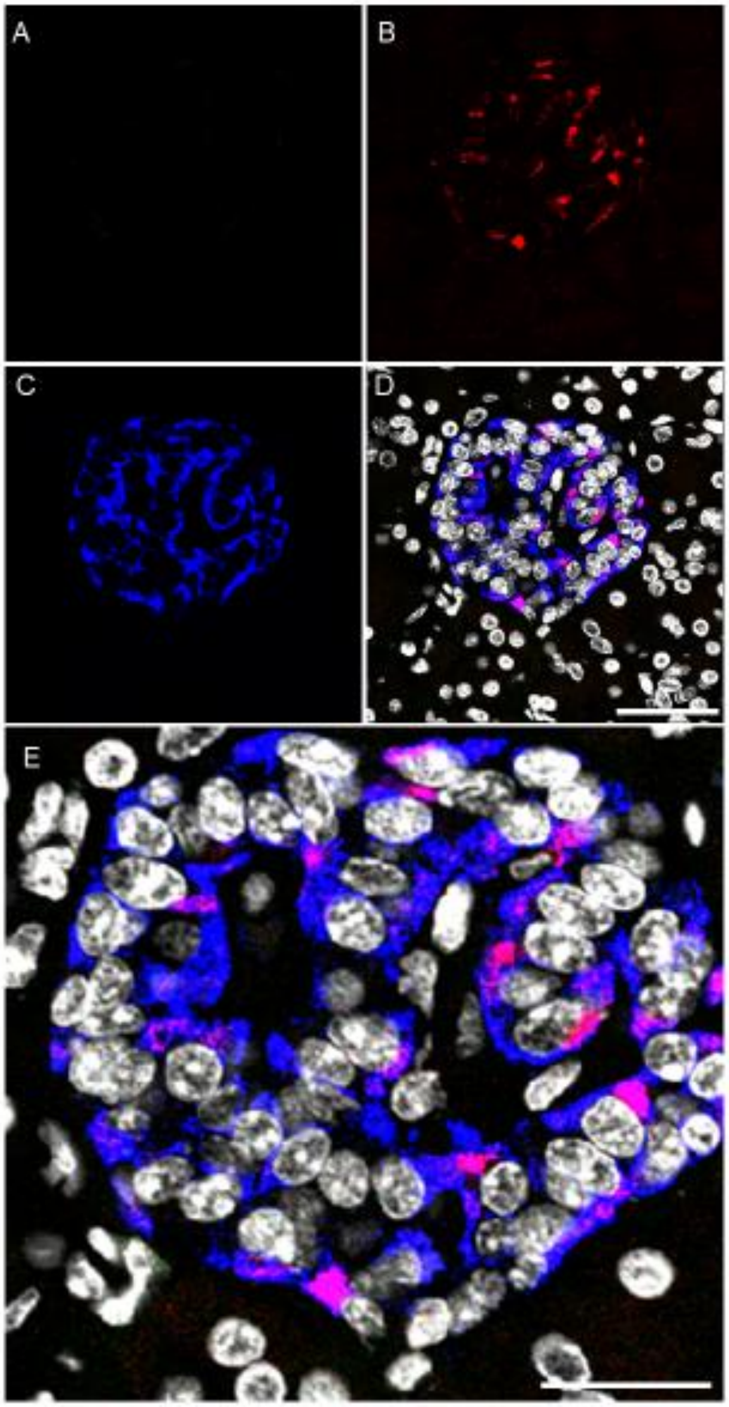

Supplementary Figure 7. CXCL10 expression distribution in IDI. Example of an IDI positive for CXCL10. The expression of CXCL10 [panel B: CXCL10 (red)] perfectly overlapped with glucagon [panel D: glucagon (blue)]. Panel A: insulin (green); panel E: colocalization CXCL10-glucagon in magenta. Scale bar panel D: 30 µm. Scale bar panel E: 20 µm.

1.2 Supplementary Tables

Supplementary Table 1. Primary and secondary antibodies and reagents used in immunostaining experiments.

Reagent	Company	Cat No	Clone	Species	Conjugate	Reactivity	[Stock]	Dilution	Incubation time	Incubation Buffer
Polyclonal Insulin	Dako	A0564		Guinea-pig	n/a	Human, mouse, rat	26,1 g/l	1:500	1h	PBS/3% BSA (Bovine serum albumin)
Polyclonal IP10 (Cxcl10)	Peprotech	500-P93		Rabbit	n/a	Human, mouse	0,1 mg/ml	1:100	O/N	PBS/3% BSA (Bovine serum albumin)
Monoclonal Glucagon	R&D System	MAB1249	181402	Mouse	n/a	Human, mouse	0,2 mg/ml	1:300	1h	PBS/3% BSA (Bovine serum albumin)
Monoclonal CD45	Dako	IR751	2B11+ PD7/26	Mouse	n/a	Human			45min	
Goat α-guinea-pig IgG (H+L) 647	Life Technologies	A21450		Goat	Alexa Fluor ® 647	Guinea pig	2 mg/ml	1:500	1h	PBS
Goat α-guinea-pig IgG (H+L) 488	Life Technologies	A11073		Goat	Alexa Fluor ® 488	Guinea pig	2 mg/ml	1:500	1h	PBS
Goat α-mouse IgG (H+L) 488	Life Technologies	A11029		Goat	Alexa Fluor ® 488	Mouse	2 mg/ml	1:500	1h	PBS
Goat α-mouse IgG (H+L) 647	Life Technologies	A21236		Goat	Alexa Fluor ® 647	Mouse	2 mg/ml	1:500	1h	PBS
Goat α-rabbit IgG (H+L) 594	Life Technologies	A11037		Goat	Alexa Fluor ® 594	Rabbit	2 mg/ml	1:500	1h	PBS
Swine α-rabbit IgG (H+L) HRP	Dako	P0217		Swine	Horseradish peroxidase (HRP)	Rabbit		1:100	1h	PBS
4′,6-Diamidino-2-phenylindole dihydrochloride (DAPI)	Sigma Aldrich	D8517					20 mg/ml	1 mg/ml	5min	PBS
TSA ® Plus TMR	PerkinElmer	NEL742001KT			Tetramethylrhodamine (TMR)				10min	
